# The clinical and CMR Characteristics of Hypertrophic Cardiomyopathy with Restrictive Phenotype

**DOI:** 10.1186/1532-429X-18-S1-P293

**Published:** 2016-01-27

**Authors:** Minjie Lu, Bailing Wu, Yan Zhang, Peter Kellman, Shihua Zhao

**Affiliations:** 1 Magnetic Resonance Imaging, Fuwai Hospital, Beijing, China; 2 Radiology, the Second Hospital of Hebei Medical University, Shijiazhuang, China; 3 Cardiovascular and Pulmonary Branch, National Heart, Lung and Blood Institute, National Institutes of Health, US Department of Health and Human Services, Bethesda, MD USA

## Background

To investigate the MR characteristics of hypertrophic cardiomyopathy (HCM) with restrictive phenotype.

## Methods

19 patients of HCM with obviously restrictive characteristics and 19 typical patients with non obstructive HCM, matched with age and gender, were retrospectively collected. The clinical features, MR morphological characteristics, and the function parameters of the two groups were compared. The paired sample test and Fisher's exact probability method were used for statistical analysis.

## Results

Patients of HCM with restrictive phenotype have more severe clinical symptoms including sustained artrial fibrillation, pericardial effusion, and worse heart function classifications compared with controls; The left and right atrium diameter were 55.79 ± 5.34 mm and 61.33 ± 11.05 mm on MR, which were significantly greater than the controls (p < 0.001); The segments with late gadolinium enhancement were 7.68 ± 2.98 in patients with restrictive phenotype, which were significantly greater than controls (5.10 ± 2.77, p = 0.008). The left ventricular end-diastolic volume index, the cardiac index, and the left heart ejection fraction of patients with restrictive phenotype were all significantly less than those of the controls.

## Conclusions

Restrictive phenotype is a special manifestation of HCM. The MR features include mild-to-moderate left ventricular hypertrophy, huge atriums, normal or small ventriculars, pericardial effusion and a wide range of late gadalinum enhancement(LGE). These patients often have severe clinical symptoms and poor prognosis. MRI has important application value in the diagnosis of the disease.

